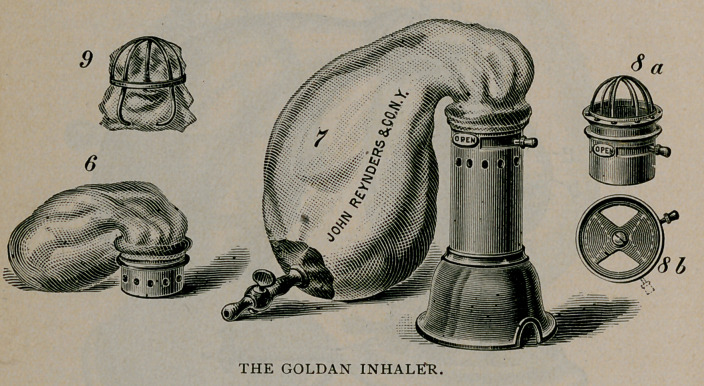# Anesthesia by Nitrous Oxide Gas and Ether1Read before the Surgical Section, Buffalo Academy of Medicine, June 5, 1900.

**Published:** 1900-09

**Authors:** Prescott Le Breton

**Affiliations:** Buffalo, N. Y.; Assistant Attending Surgeon to the Buffalo Hospital of the Sisters of Charity, 525 Delaware Avenue


### ANESTHESIA BY NITROUS OXIDE GAS AND ETHER.[1]

By PRESCOTT LE BRETON, M. D., Buffalo, N. Y.
Assistant Attending Surgeon to the Buffalo Hospital of the Sisters of Charity.

THE past two years have seen a marked change in the method of giving anesthetics in New York City, and now anesthesia by "gas and ether" is in common use in the large hospitals there. For over two years it has been employed steadily in Roosevelt for all cases, also for the past year in the New York, and for six months at the Presbyterian and St. Luke's Hospitals. At Bellevue, however, it has been given only in selected cases during the past year. Considering these facts it will be most interesting to inquire into the history of this method, the means of administration and its advantages and disadvantages.

Anesthesia by nitrous oxide gas and ether is not new. Clover, in England, in 1876, was the originator of the process and of the first

1. Read before the Surgical Section, Buffalo Academy of Medicine, June 5, 1900.

form of apparatus, and it is in England that we find the majority of the defenders of this form of anesthesia. By 1896, it was in constant use in London at the larger hospitals. In 1896, Robert Weir, at the New York Hospital, gave gas and ether to about thirty-five cases, but discontinued the administration on account of the cyanosis produced by the gas. Later he resumed its use. In the latter part of 1897, Dr. Thomas L. Bennett came to New York City, and called the attention of the leading surgeons strongly to Clover's method. He was appointed anesthetist to the Hospital for Ruptured and Crippled.

THE HEWITT INHALER.

McBurney became interested in Bennett's work, and during the first part of 1898, the writer, while house-surgeon, had the good fortune of seeing gas and ether given at Roosevelt, about 200 times. Recently Drs. Mann and Crockett have begun to anesthetise cases in this manner at the Buffalo General Hospital.

The chief object of this method of preceding the administration of ether by gas is the anesthetising of the patient quickly without the usual "stage of excitement," to pass to full anesthesia under ether inside of four minutes, often in two to three minutes. Nitrous oxide is nonirritating and nonstimulating, rapid in action, and gives the patient no sensation of impending suffocation. Chloral, alcohol, morphine and local applications to the upper air passages have their objectionable features when used to lessen the unpleasant effects at the start of etherisation. Likewise is it so when chloroform or the A. C. E. mixture is substituted. Nitrous oxide, however, is an improvement as a precedent to etherisation. Practically its only contraindication is an atheromatous condition of the arteries.

The favorite apparatus in New York City is the Bennett modification of the Hewitt inhaler, made by Kny-Scheerer ($40.00.) The Hewitt inhaler ($30.00) is more bulky. These forms permit the turning on of the ether while the gas is still being given. It is less

THE BENNETT INHALER.

expensive to have two inhalers, one, the usual gas inhaler found at the dental establishments and the other the Ormsby inhaler.

The apparatus now used by Dr. Mann is recommended on account of its simplicity, cheapness and serviceability. It was devised by Dr. Goldan, and is made by Reynders ($13.00.) It differs from all others in that it dispenses with valves and yet allows ether to be given with the gas before the narcosis is complete. It consists of a face-piece of rubber and a metal cylinder, holding a piece of gauze for the ether. Into the end of the cylinder can readily be fitted the usual gas attachment. Later this gas attachment is removed and a rubber bag adjusted, which with the cylinder acts as an Ormsby inhaler.

Before administering the gas the same precautions are taken as before any etherisation. If the Hewitt inhaler is employed, after the intaking of six to ten breaths of pure gas, ether is gradually added and the gas as gradually turned off until ether alone is being inhaled. With the Goldan apparatus the following method is advisable. The rubber bag for the gas is emptied of air and filled with gas from the cylinder. The face piece is closely adjusted to the patient, who is told to breathe deeply and regularly. In about twenty seconds, when the sensation in the glottis is dulled the cylinder and gas attachment are disconnected, one drachm of ether is poured on the gauze in the cylinder and the gas attachment readjusted speedily. The patient now breathes gas and ether simultaneously. In a few seconds another drachm of ether is poured into the cylinder and later another drachm.

THE GOLDAN INHALER.

At the end of one minute or thereabouts, certain signs appear, a change in the rhythm of respiration, *i. e.*, from full deep regular breathing to rapid and somewhat jerky breathing, a slight cyanosis ensues and the patient can no longer voluntarily hold up his arms. The gas attachment is now withdrawn, the rubber bag is fitted to the cylinder and ether alone administered in drachm doses, occasionally repeated.

The disadvantage in using the ordinary dental gas inhaler and then changing to the Ormsby, which is an old plan, is that choking and coughing frequently follow, as there is no gradual transition from gas to ether, as allowed by either the Bennett or the Goldan apparatus, but a sudden and dense ether vapor is presented to the patient who has been breathing gas. The same signs are watched for, a change in the rhythm of the breathing and a slight cyanosis. Then at the end of an inspiration the dental inhaler is taken away and the Ormsby containing the ether is adjusted. The patient frequently coughs and sometimes it is necessary to allow one or more inspirations of pure air, or respiration stops and the cyanosis deepens. To avoid this mishap it is well to have the air-valve of the Ormsby open at first, that the ether vapor may be diluted. Patients differ as to the relative amount of air required while breathing into a "close" inhaler, but in general more air is needed the longer the patient is unconscious.

There are several practical points to be remembered and emphasised in the administration. First of all speed is essential in the transition from gas to ether, that no time is lost and that as little air as possible is given. The pulse may be disregarded throughout these first few moments. The face piece of the gas inhaler must be closely adjusted to exclude air. The commonest error is to continue the administration of the gas until muscular twitching, deep cyanosis, stoppage of respiration and dilatation of pupils occur. This stage must never be reached or consciousness will be resumed before the anesthetiser succeeds in establishing normal respiration.

The advantages are:

1. Comfort to the patient. All cases object strenuously to being anesthetised by ether alone after experience with this method.

2. Freedom from excess of mucus and saliva as the glands are not stimulated by the gas.

3. Less ether is required. Two ounces are ordinarily sufficient for a major operation.

4. Diminished chance of kidney complications, owing to the small amount of ether inhaled.

5. Cheapness. After the apparatus is paid for nitrous oxide gas and ether are less expensive than ether alone.

6. Alcoholics are anesthetised with less trouble by this method than any other according to some authorities.

7. The amount of time saved for the surgeon when several operations follow each other is a weighty consideration.

8. The mastering of this method is not difficult. A hospital interne can ordinarily learn how to administer gas and ether satisfactorily after two or three attempts.

9. Frederick Treves gives gas and ether to patients of all ages, male or female.

10. Safety. In the literature which the writer has seen there is the report of but one death. Anesthetists of large experience, as

Hewitt, Braine, Bailey, Bennett and Goldan agree as to the safety of this means of inducing narcosis.

The chief disadvantage is the cost and bulkiness of the apparatus. Some writers dislike the "close" inhaler, as the patient necessarily inspires again and again his own breath, including the carbon dioxide and particles of organic matter. It is questionable if this objection is not of theoretical rather than practical value.

BIBLIOGRAPHY.

1. Hewitt, Braine, Bailey and Treves. *The Practitioner*, October, 1896.
2. Bennett. *Medical Record*, Vol. LIII., No. 9, p. 296.
3. Gibney and Weir *Medical Record*, Vol LIII., No. 13, p. 457.
4. Goldan. *Journal of the American Medical Association*, March 24, 1900.
5. *Indiana Medical Journal*, May 10, 1900, p. 451.
6. Goldan. *Medical Record*, April 14, 1900.

525 Delaware Avenue.

## Figures and Tables

**Figure f1:**
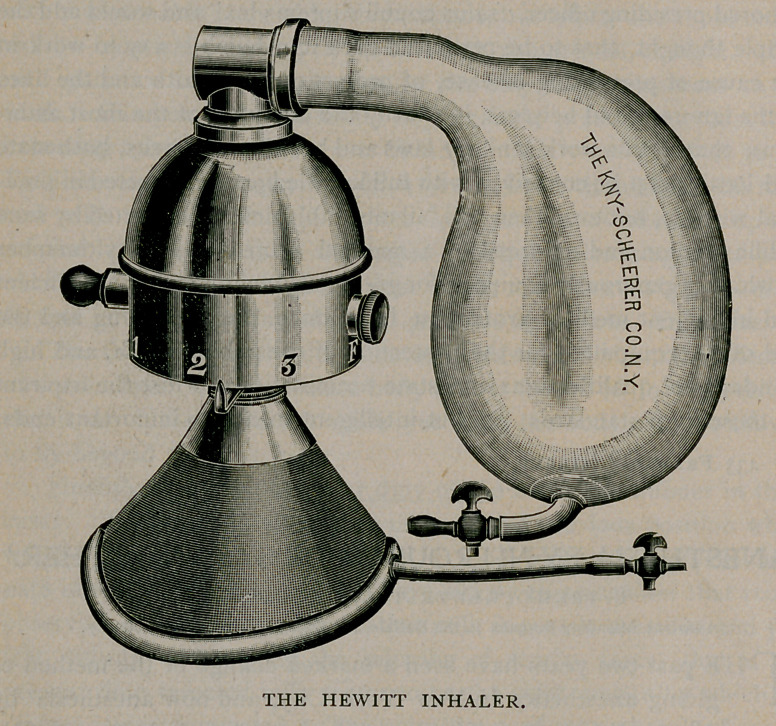


**Figure f2:**
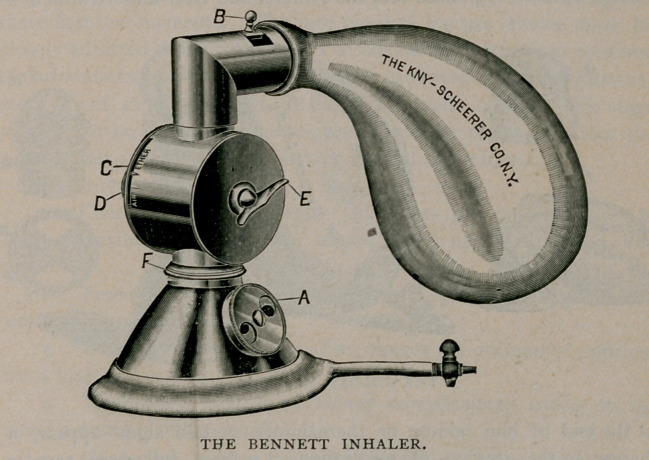


**Figure f3:**